# Sintilimab and Chidamide for Refractory Transformed Diffuse Large B Cell Lymphoma: A Case Report and A Literature Review

**DOI:** 10.3389/fonc.2021.757403

**Published:** 2021-11-08

**Authors:** Chao Chen, Wei Zhang, Daobin Zhou, Yan Zhang

**Affiliations:** Department of Hematology, Peking Union Medical College Hospital, Peking Union Medical College, Chinese Academy of Medical Sciences (CAMS), Beijing, China

**Keywords:** anti-PD-1, HDAC inhibitor, transformed diffuse large B cell lymphoma, histologic transformation, refractory

## Abstract

Patients with relapsed/refractory (R/R) transformed diffused large B cell lymphoma (tDLBCL) have a poor prognosis and a low survival rate. In addition, no standard therapy has yet been established for R/R tDLBCL. Herein we presented a single case of a patient with R/R tDLBCL who was successfully treated with sintilimab and chidamide. The patient was a 71-year-old man with pulmonary mucosa-associated lymphoid tissue lymphoma. He did not receive any treatment until tDLBCL was confirmed 2 years later. The tDLBCL was primary refractory to R2-CHOP, R2-MTX, and Gemox regimens. However, the patient achieved sustained complete remission after the combination therapy of sintilimab and chidamide. To the best of our knowledge, this is the first report of sintilimab combined with chidamide for the treatment of R/R tDLBCL, which opens up new therapeutic possibilities for this new combination therapy in future prospective clinical trials.

## Introduction

Mucosa-associated lymphoid tissue (MALT) lymphoma is an indolent lymphoma that generally has a favorable prognosis and slow progression. Patients with MALT lymphoma have a median overall survival (OS) of 12.6 years (1). However, the histologic transformation (HT) of MALT lymphoma may lead to a more aggressive phenotype, such as diffused large B cell lymphoma (DLBCL) (2), yet the frequency of transformation in indolent lymphoma is low (3). Patients with DLBCL have a poor prognosis and a reduced 5-year OS from 86 to 65% compared to non-transformed MALT lymphoma (4). Moreover, there are no standard regimens for DLBCL. R-CHOP regimen is the most commonly used therapy for tDLBCL (5), while the complete remission rates of R-CHOP in DLBCL and *de novo* DLBCL are 65 and 75%, respectively, with no significant differences (6). Regrettably, relapsed/refractory (R/R) transformed DLBCL (tDLBCL) is not included in most clinical trials for R/R indolent B-cell non-Hodgkin lymphoma or R/R *de novo* DLBCL, resulting in the lack of evidence on novel treatments. Recently, some novel agents, such as lenalidomide and ibrutinib, have demonstrated moderate efficacy in R/R tDLBCL with manageable adverse events (7, 8), yet the efficacy of current therapies is still unsatisfactory.

Chidamide is a histone deacetylase inhibitor (HDACi) approved for the treatment of R/R peripheral T-cell lymphoma in China. Recent studies have shown that chidamide can induce apoptosis in the DLBCL cell line (9), enhance immune cell-mediated tumor cell cytotoxicity, and show board anti-tumor activity (10). Programmed cell death protein 1 (PD-1) antibody, an agent that targets the PD-1 immune checkpoint, has become a promising therapeutic approach in many types of tumor (11). However, the PD-1 antibody has an unsatisfactory efficacy in R/R DLBCL as a single agent. A phase II study on nivolumab monotherapy in R/R DLBCL showed that the overall response rates (ORRs) were 3 and 10% in autologous stem cell transplantation (ASCT)-ineligible and ASCT-failed cohorts (12), respectively.

Recently, several preclinical studies have revealed that HDACi could enhance immune cell activity and augment response to PD-1 antibody. The combination of HDACi and PD-1 antibodies can synergically enhance the anti-tumor activity in different types of tumor (13, 14). Herein we reported a case of concurrent application of sintilimab and chidamide for R/R tDLBCL.

## Case Presentation

In February 2018, a 71-year-old male patient without previous history of pulmonary disease complained of dull pain in the left chest region; the pain expanded to the left axilla. A computed tomography (CT) scan of the chest showed multiple patchy ground-glass shadows, with diffuse dotted hyperdense shadows, in the bilateral lungs and multiple small lymph nodes in the mediastinum. The patient was diagnosed with MALT lymphoma based on the pathological analysis of the right pulmonary core needle biopsy. Immunohistochemical analysis of the biopsy tissue revealed the following results for lymphoma cells: CD3(-), CD20(+), Bcl-2(+), CD23(-), CD10(-), Bcl-6(-), CD5(-), Cyclin D1(-), Mum-1(-), and Ki-67 (10%). Moreover, a positron emission tomography/CT (PET/CT) scan showed increased fluorodeoxyglucose (FDG) uptakes in multiple patchy hyperdense shadows of the bilateral lungs and a massive shadow of the medial basilar segment of the right lower lobe. The patient was managed by a watch-and-wait strategy considering the lack of clear indications for treatment.

**Figure 1:**
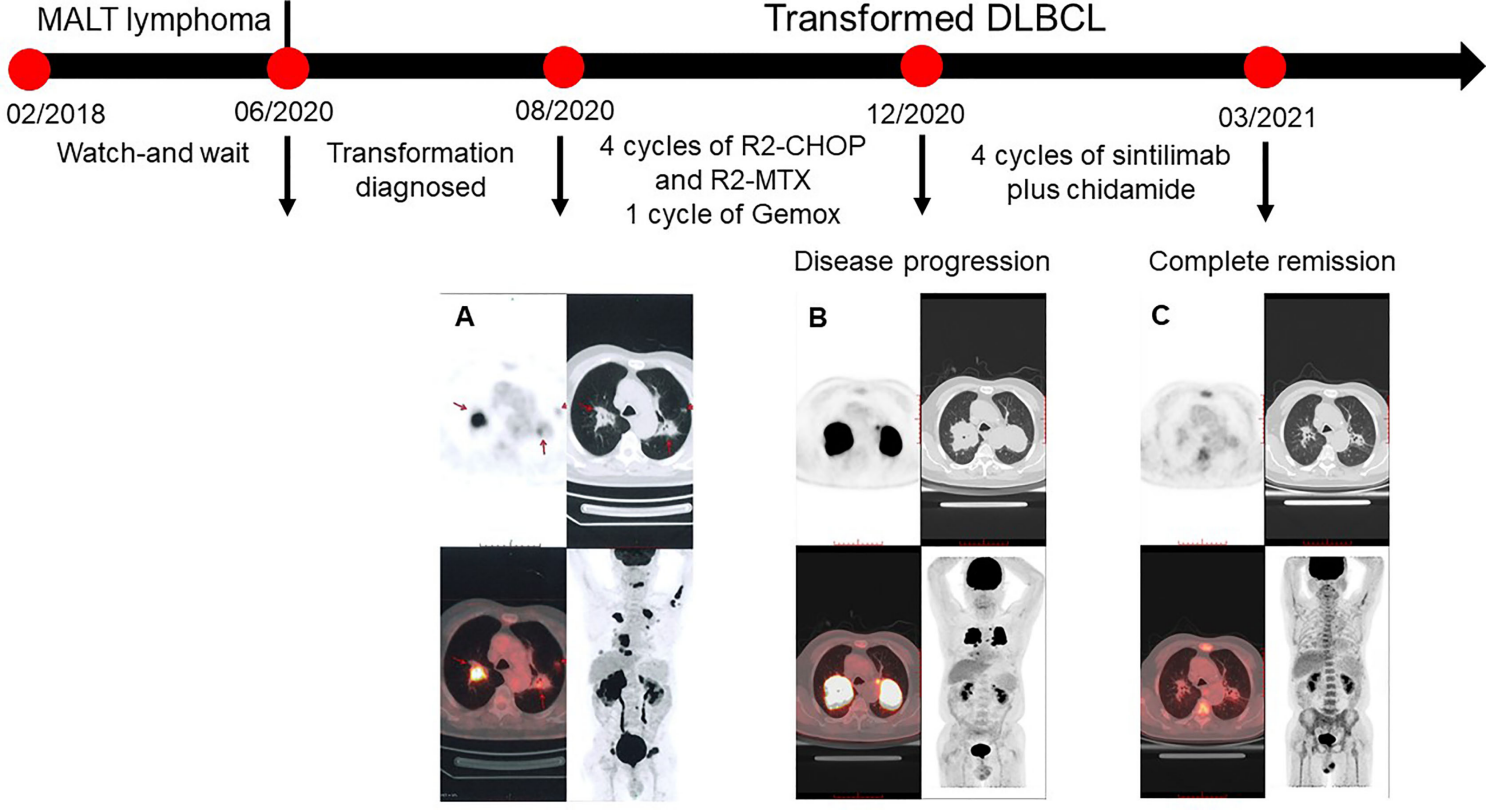
PET/CT scans of the patient. **(A)** Baseline. The fluorodeoxyglucose (FDG) uptakes increased in multiple patchy shadows and massive consolidations of the bilateral lungs (SUVmax = 22.1), multiple partial bones (SUVmax = 19), multiple swelling lumbosacral nerve roots (SUVmax = 16), masses in the bilateral adrenal glands (SUVmax = 31.2), and a nodule (0.7 cm) between cervical rear muscles. **(B)** Disease progression after first-line chemotherapy. The sizes and FDG uptakes of massive consolidations in the bilateral lungs increased with SUVmax of 31.5. FDG uptakes of other lesions decreased. **(C)** The patient achieved a complete response after four cycles of sitilimab and chidamide. The SUVmax of lesions in the left and right lungs were 2.4 and 2.1, which indicated a Deauville score of 2.

In June 2020, the patient was referred to our hospital because of continuous pain in the back of the right thigh. The pain gradually expanded to the right leg and planta, accompanied by numbness. The patient could not stand, and pitting edema started to gradually appear in the right lower limb. The physical examination showed that the patient had peripheral facial paralysis, with an Eastern Cooperative Oncology Group score of 3. The PET/CT re-examination (**Figure 1A**) revealed increased FDG uptakes in multiple patchy shadows and massive consolidations in the bilateral lung, bilateral adrenal glands, multiple bones, and lumbosacral nerve roots. The adrenal mass biopsy tissue was obtained by core needle biopsy. Based on the pathological analysis, the patient was diagnosed with DLBCL, a non-germinal center B cell-like (non-GCB) subtype. The immunohistochemical analysis of the biopsy tissue showed the following results for lymphoma cells: CD20(+), Bcl-2(+), CD23(-), CD10(-), Bcl-6(+), CD5(-), Cyclin D1(-), Ki-67 (70%), Mum-1(+), c-Myc (<10%+), and SOX11(-). The fluorescence *in situ* hybridization test yielded negative results for EBER ISH, MYC, and BCL2/IGH. The lumbar puncture examination yielded elevated cerebrospinal fluid (CSF) pressure (205 mmH_2_O), protein concentration (0.9 g/L, normal reference value: 0.15–0.45 g/L), and interleukin-10 concentration (235.0 ng/L, normal reference value: 4.3–6.9 ng/L). The cytological examination of CSF showed some atypical cells. In addition, the flow cytometry examination of the CSF revealed that kappa-restricted aberrant B cells constituted 13.1% of karyocytes, which indicated central nervous system involvement. Circulating free DNA of DLBCL testing *via* next-generation sequencing (Illumina) detected the following mutations: *BCL6* (2.8%), *TNFRSF* (1.36%), *CREBBP* (0.9%), *PIM1* (0.8%), *MEF2B* (0.6%), *BCL2* (0.6%), and *MALT1-BIRC3* fusion (0.3%). The result of the liver and kidney function examination and blood routine tests were roughly normal, with a normal concentration of lactic acid dehydrogenase. According to his previous MALT lymphoma history, the patient was diagnosed with transformed DLBCL (Ann Arbor IVB stage and International Prognosis Index of 4), which had extensive extra-nodal involvements and central nervous system involvements.

**Figure 2:**
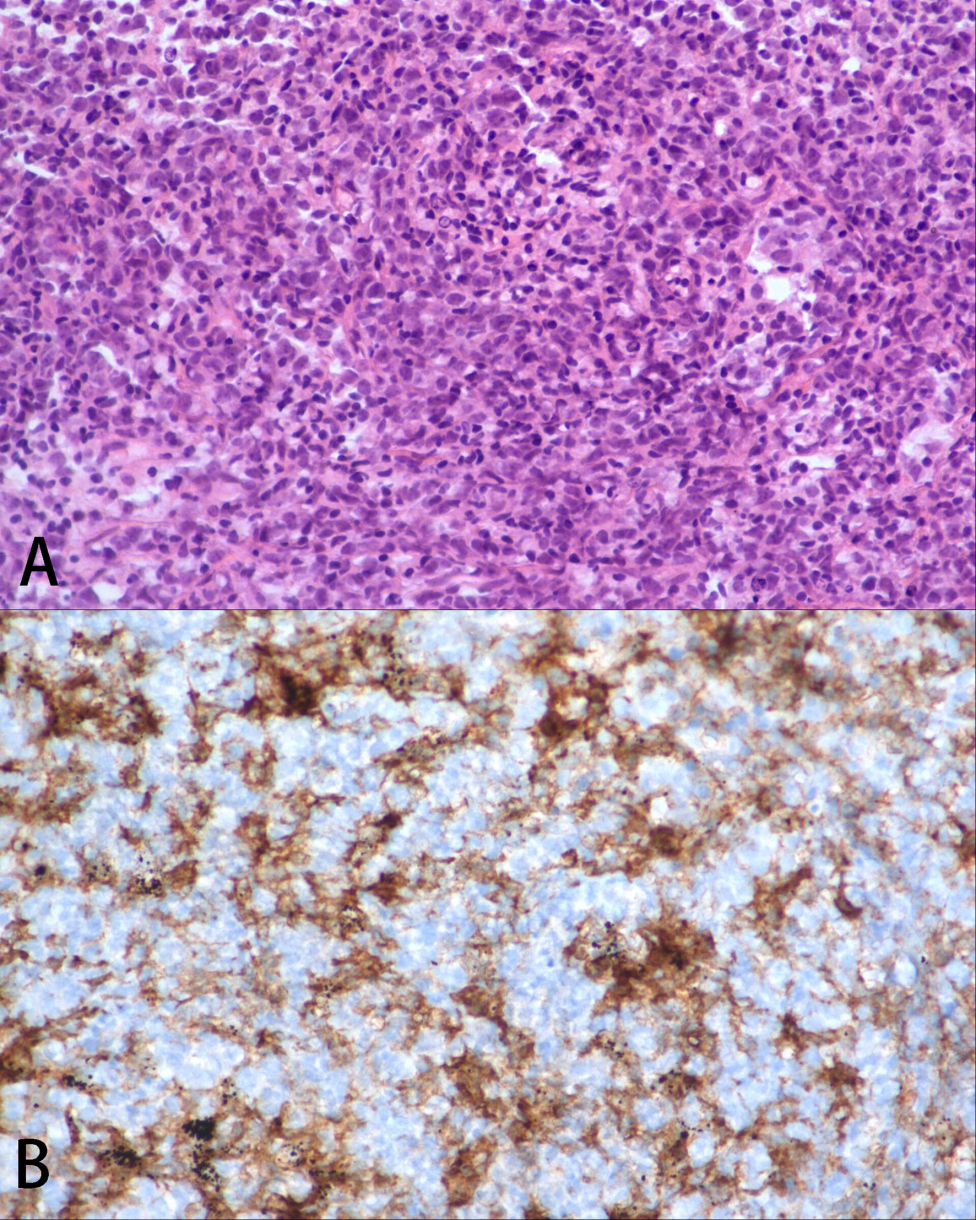
Pathological analysis. **(A)** Pulmonary histopathology at disease progression, which was consistent with diffused large B cell lymphoma, ×200 H&E. **(B)** The lymphoma cells were PD-L1 positive, ×200.

In August 20, 2020, due to systemic and central nervous system lesions, the patient received four cycles of R2-CHOP (rituximab, lenalidomide, cyclophosphamide, epirubicin, vincristine, and prednisone) and R2-high-dose MTX (rituximab, lenalidomide, and methotrexate) alternative regimens as first-line therapy. The patient also received intrathecal injections of methotrexate (10 mg), dexamethasone (5 mg), and cytarabine (50 mg) during each cycle. However, after four cycles of treatment, an interim PET/CT scan (**Figure 1B**) revealed that the sizes and FDG uptakes of the pulmonary and mediastinal lesions progressed (SUVmax = 31.5), with significantly reduced sizes of the other lesions. A lumbar puncture re-examination showed decreasing CSF pressure (180 mmH_2_O) and protein concentration (0.66 g/L). Based on the interim examination, pulmonary and mediastinal lesions revealed the progress of the disease, although the central nervous system and other peripheral involvements were in remission. The pulmonary core needle biopsy confirmed the presence of lymphoma. On December 20, 2020, the therapy was switched to one cycle of Gemox regimen (gemcitabine and oxaliplatin) as a second-line therapy, but the lesions of the bilateral lungs that were still present suggested the progression of the disease. There was no preferred regimen for primary refractory tDLBCL. A repeat pathological analysis biopsy showed that the lymphoma was still DLBCL with PD-L1 expression (**Figure 2**). The patient also had a mutation of *CREBBP*. Previous studies revealed that PD-L1 expression was associated with PD-1 antibody sensitivity and that mutation in *CREBBP*, an acetyltransferase, indicated that HDACi may be effective (15, 16). Therefore, the patient received sintilimab plus chidamide as salvage therapy. The dose of sintilimab was 200 mg intravenously once per cycle, and the dose of chidamide was 30 mg orally twice a week, with the courses repeated every 21 days. After four cycles of combination treatment, a PET/CT scan (**Figure 1C**) showed that the patient was considered to be in complete metabolic remission according to the 2014 Lugano criteria. A lumbar puncture confirmed the sustained clearance of lymphoma.

During the combination therapy, the patient experienced adverse events that were manageable. In the third cycle of treatment, the patient developed thrombocytopenia (50 × 10^9^/L), and the dose of chidamide was adjusted to 20 mg orally twice a week. In the fourth cycle, he also developed grade 2 neutropenia (1.18 × 10^9^/L), which was also considered to occur due to chidamide. No non-hematological adverse reactions, such as gastrointestinal symptoms, rash, malaise, and liver and kidney damage, and no anemia or immune-related adverse reactions occurred during the treatment. At the time of this writing, the patient was in the ninth cycle of the sintilimab and chidamide combination treatment, without any symptoms or evidence of disease progression.

## Discussion

MALT lymphoma is an indolent lymphoma. Indolent lymphoma can undergo HT into an aggressive type, primarily DLBCL, with a poorer prognosis and shorter OS (5-year rate, 65 *vs*. 86%) (4). Because HT is often excluded from most clinical trials and has a low prevalence, there are few studies on new treatment options for tDLBCL.

In this case, the patient with tDLBCL received R2-CHOP and R2-MTX regimens as first-line therapy, but his disease progressed. After switching to the Gemox regimen as second-line therapy, the patient remained refractory. R/R DLBCL has a poor prognosis. In the SCHOLAR-1 study, R/R DLBCL patients treated with chemotherapy regimens showed an ORR of 26%, complete response (CR) rate of 7%, and a median OS of 6.2 months (17). Like R/R DLBCL, R/R tDLBCL also has a poor prognosis, likely due to acquired chemoresistance and host features, including immunoparesis and poor treatment tolerance (7). Currently, there are no standard treatments for R/R tDLBCL, and patients are usually treated following a similar approach as that for R/R DLBCL.

After the failure of second-line therapy, our patient received sintilimab and chidamide combination treatment and achieved sustained complete remission. To the best of our knowledge, this case report is the first report on sintilimab and chidamide combination treatment for R/R tDLBCL, which showed that this therapy not only achieved an excellent short-term outcome without a severe adverse reaction but also had the possibility of durable efficacy. The combination therapy of sintilimab and chidamide may be used as a potential therapy for R/R tDLBCL.

Several preclinical studies revealed that PD-1 antibody and HDACi have a synergistic effect in different mouse models (13, 18). There is mounting evidence suggesting that establishing an integrated immune cycle is needed for the effective response to PD-1 antibody therapy, and an impaired immune cycle can cause immunotherapy failure. Epigenetic modification can restore the impaired immune cycle by reprogramming the tumor microenvironment, increasing tumor antigen presentation, and regulating T cell activity (14). Recent studies of different tumor models revealed that a combination of HDACi and PD-1 antibodies could elicit changes in the tumor microenvironment, such as increasing the infiltration of CD4^+^ T cells, CD8^+^ T cells, and central and effector memory T memory cells and decreasing the infiltration of M2 macrophages, myeloid-derived suppressor cells, and Treg cells (18–21). In terms of antigen presentation, HDACi can reverse low HLA class I expression. A recent case report suggested that the HDACi and PD-1 antibody combination may increase antigen presentation *via* HLA class I and enhance T-cell infiltration (22, 23). Referring to the regulation of T cell activity, HDACi can also induce IL-2 and IFN-γ expression and increase the proliferation of CD8+ T cells and NK cells while inhibiting the proliferation of Treg cells, which promotes the anti-tumor effect of PD-1 antibody (18). In addition to these observations, a study in colon cancer has indicated that HDACi romidepsin upregulated the expression of PD-L1 in cancer cells and enhanced the anti-tumor effect of anti-PD-1 therapy (24). These observations exhibit part of the synergistic effect of sintilimab and chidamide, yet the underlying mechanisms of action remain unclear. Thus, more studies are needed to explore the mechanism of the combination of sintilimab and chidamide therapy in R/R tDLBCL.

As a single agent, HDACi and PD-1 antibodies show modest efficacy in clinical studies of R/R DLBCL. In a meta-analysis that studied 114 patients with non-Hodgkin lymphoma treated with nivolumab or pembrolizumab, the ORR of PD-1 antibody monotherapy was 30.77% in patients with R/R NHL (25), which included 82 patients with DLBCL. In a phase II study of nivolumab in R/R DLBCL patients who were ineligible for or had failed autologous hematopoietic cell transplantation (auto-HCT), ORR, median progression-free survival (PFS), and OS were 10%, 1.9 months, and 12.2 months in the auto-HCT-failed cohort and 3%, 1.4 months, and 5.8 months in the auto-HCT-ineligible cohort (12), respectively. Furthermore, two phase II trials of HDACi in R/R DLBCL showed that the ORR of mocetinostat was 18.9% in 41 patients, and the ORR of panobinostat was 28% in 40 patients (26, 27). Although HDACi and PD-1 antibody have modest efficacy as a single agent, some published literature proved that PD-1 antibody and HDACi had synergistic effects in different kinds of tumor. Two case reports on NK/T cell lymphoma have reported that combined sintilimab and chidamide therapy may lead to successful outcomes (28, 29). Moreover, a phase IB/II trial that included 38 patients with R/R extranodal NK/T cell lymphoma who were treated with sintilimab plus chidamide showed an ORR of 59.5% and a CR rate of 48.6% (30). Although HDACi plus PD-1 antibody treatment is rarely studied in lymphoma, it is often studied in head and neck squamous cell carcinomas, salivary gland cancer, non-small cell lung cancer, and other types of cancer (31, 32). In these studies, this combination therapy revealed effective anti-tumor activity despite progression on prior immune checkpoint inhibitor treatment. Thus, we treated the patient with sintilimab and chidamide. The combination of sintilimab with chidamide therapy for R/R tDLBCL led to a successful outcome in this case, even after multiple cycles of prior therapy. These findings suggest that the impressive outcome may be due to the synergistic effect of sintilimab and chidamide. The combination of sintilimab and chidamide may overcome the drug resistance of a single agent.

In this case, the patient experienced only hematologic adverse events, including thrombocytopenia and neutropenia, without immune-related adverse events and severe adverse events. Compared to the previous safety profiles of a phase I/Ib study of PD-1 antibody pembrolizumab plus HDACi vorinostat for non-small cell lung cancer and a phase Ib/II trial of sintilimab plus chidamide for NK/T cell lymphoma, the treatment-related adverse events of this case were consistent with the observations of previous findings, without additional toxicity appearing (28, 30, 31). These findings indicated that the combination of sintilimab and chidamide has a favorable safety profile.

In this report, the immunohistochemical analysis showed that PD-L1 expression was positive in 15% of lymphoma cells. A previous study demonstrated that pembrolizumab plus R-CHOP could improve the PFS and CR rate of R-CHOP in DLBCL patients with PD-L1 expression, which contradicted the historical data in R-CHOP-treated patients with PD-L1 expression (33). Combining the previous study with this report, these findings support the idea that DLBCL patients with PD-L1 expression may be more likely to benefit from sintilimab.

DNA sequencing of tumor tissue is a usual and useful tool for diagnosis, disease monitoring, and mutational profile detection in different types of tumor, including DLBCL (34–36). In recent years, development in the detection and sequencing of ctDNA from peripheral blood provided a noninvasive approach for tumor genetic diversity analysis and clinical decision-making (37–39). In DLBCL, targeted next-generation sequencing of ctDNA could detect at least one mutation in 63 to 100% patients. The sensitivity was more than 90%, and the specificity was 100% (26, 40–42). The concordance of mutations between plasma and matched tumor samples was estimated to be between 65 and 98% in retrospective studies (43). In addition to mutational profile, several studies have revealed the potential of ctDNA in predicting prognosis and monitoring relapse in DLBCL (42, 44, 45). Kurtz et al. studied the dynamics of ctDNA from 217 patients with DLBCL and used a training and validation framework. The result showed that the pretreatment levels were prognostic in both front-line and salvage settings and that molecular responses were also independent prognostic of outcomes (46). In this case, ctDNA sequencing also showed that the patient had *CREBBP* mutation. The *CREBBP* gene encodes a histone acetyltransferase, and its loss-of-function mutation is among the most common genetic alterations in DLBCL. In small cell lung cancer, follicular lymphoma, and DLBCL, *CREBBP* loss reduces histone acetylation and transcription of cellular adhesion genes while driving tumorigenesis. HDACi can restore these effects, restore the immune ability of tumor-infiltrating lymphocytes in an MHC class I and II-dependent manner, and even synergize with PD-L1 blockade in a syngeneic model *in vivo*, which shows enhanced anti-tumor effectiveness in tumors with *CREBBP* mutation (16, 47, 48). Considering that the patient with *CREBBP* mutation in this case also benefited from HDACi, *CREBBP* mutation may be a potential biomarker of chidamide sensitivity and even a biomarker of chidamide plus sintilimab sensitivity.

This study has a few limitations. This is a retrospective case report that lacks basic research. Based on this single case, drawing a strong conclusion is inappropriate. Based on our knowledge, there are no clinical studies on the combination of sintilimab and chidamide for R/R tDLBCL or DLBCL. Thus, to explore the efficacy and mechanism of this combination treatment, a more detailed and well-designed prospective study is needed in the future.

In conclusion, this case report suggested that a combination of sintilimab and chidamide may be used to treat patients with R/R tDLBCL, yet prospective clinical trials are needed to prove the impressive efficacy and favorable safety profile of sintilimab combined with chidamide in R/R tDLBCL in the future.

## Data Availability Statement

The datasets presented in this article are not readily available due to ethical and privacy restrictions. Requests to access the datasets should be directed to the corresponding author.

## Ethics Statement

Written informed consent was obtained from the individual(s) for the publication of any potentially identifiable images or data included in this article.

## Author Contributions

CC and YZ wrote the first draft of the manuscript. YZ, DZ, and WZ participated in the diagnosis and treatment of the patient and provided follow-up. CC and ZY acquired clinical data. All authors contributed to the article and approved the submitted version.

## Funding

This work was supported by the National Natural Science Foundation of China (no. 81970188), the Natural Science Foundation of Beijing Municipality (no. 20G10757), and the Teaching Reform Project of Peking Union Medical College (2019zlgc0107).

## Conflict of Interest

The authors declare that the research was conducted in the absence of any commercial or financial relationships that could be construed as a potential conflict of interest.

## Publisher’s Note

All claims expressed in this article are solely those of the authors and do not necessarily represent those of their affiliated organizations, or those of the publisher, the editors and the reviewers. Any product that may be evaluated in this article, or claim that may be made by its manufacturer, is not guaranteed or endorsed by the publisher.
